# Decreased thermal tolerance under recurrent heat stress conditions explains summer mass mortality of the blue mussel *Mytilus edulis*

**DOI:** 10.1038/s41598-019-53580-w

**Published:** 2019-11-25

**Authors:** Laurent Seuront, Katy R. Nicastro, Gerardo I. Zardi, Eric Goberville

**Affiliations:** 1 CNRS, Univ. Lille, Univ. Littoral Côte d’Opale, UMR 8187, LOG, Laboratoire d’Océanologie et de Géosciences, F 62930 Wimereux, France; 2 Department of Marine Energy and Resource, Tokyo University of Marine Science and Technology, 4-5-7 Konan, Minato-ku Tokyo, 108-8477 Japan; 3 Department of Zoology and Entomology, Rhodes University, Grahamstown, 6140 South Africa; 4 CCMAR–Centro de Ciencias do Mar, CIMAR Laboratório Associado, Universidade do Algarve, Campus de Gambelas, Faro, 8005-139 Portugal; 5 BOREA, Biologie des Organismes et des Ecosystèmes Aquatiques, UMR Muséum National d’Histoire Naturelle, Sorbonne Université, Université de Caen Normandie, Université des Antilles, CNRS 7208, IRD 207, 43 Rue Cuvier, 75005 Paris, France

**Keywords:** Climate-change ecology, Climate-change ecology, Ocean sciences, Marine biology

## Abstract

Extreme events such as heat waves have increased in frequency and duration over the last decades. Under future climate scenarios, these discrete climatic events are expected to become even more recurrent and severe. Heat waves are particularly important on rocky intertidal shores, one of the most thermally variable and stressful habitats on the planet. Intertidal mussels, such as the blue mussel *Mytilus edulis*, are ecosystem engineers of global ecological and economic importance, that occasionally suffer mass mortalities. This study investigates the potential causes and consequences of a mass mortality event of *M. edulis* that occurred along the French coast of the eastern English Channel in summer 2018. We used an integrative, climatological and ecophysiological methodology based on three complementary approaches. We first showed that the observed mass mortality (representing 49 to 59% of the annual commercial value of local recreational and professional fisheries combined) occurred under relatively moderate heat wave conditions. This result indicates that *M. edulis* body temperature is controlled by non-climatic heat sources instead of climatic heat sources, as previously reported for intertidal gastropods. Using biomimetic loggers (i.e. ‘robomussels’), we identified four periods of 5 to 6 consecutive days when *M. edulis* body temperatures consistently reached more than 30 °C, and occasionally more than 35 °C and even more than 40 °C. We subsequently reproduced these body temperature patterns in the laboratory to infer *M. edulis* thermal tolerance under conditions of repeated heat stress. We found that thermal tolerance consistently decreased with the number of successive daily exposures. These results are discussed in the context of an era of global change where heat events are expected to increase in intensity and frequency, especially in the eastern English Channel where the low frequency of commercially exploitable mussels already questions both their ecological and commercial sustainability.

## Introduction

Extreme events such as heat waves, droughts, storms and floods have increased in frequency and duration over the last decades^1–3^ and episodes considered rare today may be the norm under future climate scenarios, as expected from model predictions^4–6^. These events are likely to affect both terrestrial and marine ecosystems and cause high mortality^7,8^, deleterious impacts on populations^9,10^, reconfigurations of communities^11–13^, threaten global biodiversity and the provision of ecosystems services^14,15^, and ultimately impact socio-economic systems^16^. Natural climate variability is also noticeably superimposed onto decadal warming trends in most regions, increasing the likelihood of discrete climatic events becoming extreme or anomalous^17–19^.

Heat waves are particularly important on rocky intertidal shores, which are one of the most thermally variable and stressful habitats on the planet^20^. In these ecosystems, the consequences of heat waves can be dramatic. For instance, heat waves are responsible for the mass mortality events documented in a range of ectothermic organisms such as juvenile barnacles^21^, limpets^22–24^ and mussels^20,25–27^. In addition, mass mortalities have the capacity to drive persistent ecosystem changes^28,29^. Intertidal mussels are important ecosystem engineers through their attachment to the substrate in dense mono- and multi-layered beds that create microhabitats that remain moist and thermally benign during low tides^30,31^ and offer protection against wave action during high tides^32^. Mussel beds also increase habitat complexity, providing substrate for colonisation, trap sediment and organic particles that serve as food for small invertebrates and shelter from predation^33–35^. Therefore, although mussels may outcompete other primary-space holders such as seaweeds and other sessile invertebrates^36,37^, their bioengineering properties often enhance local biodiversity by facilitating the establishment and persistence of a variety of small invertebrates^38–40^.

Mussels are also of prime economic importance, in particular the blue mussels *Mytilus edulis* (Linnaeus, 1758) and *M. galloprovinciallis* (Lamarck, 1819). They are estimated an average net worth ranging from $2,480,000 to $102,000,000 in the United States^41^. In Europe, the culture of these species produces about 50% of the annual world-wide harvest of mussels^42^, and represent 48% of the 160,000 tons of bivalves produced annually in France^43^. Numerous natural and shellfish mussel beds are also scattered along the French coast of the eastern English Channel. The production of the latter is of industrial extent, covering 55 hectares and representing a production of 2,600 tons annually, worth 1.8×10^7^ €^44^. In turn, natural mussel beds, though professionally exploited and representing ca. 450 tons per year^44^, are also traditionally exploited by locals who are allowed to take up to 5 litres of mussels larger than 4 cm in length per day, a recreational fishery worth ca. 250 tons per year^45^.

Despite an increase in mussel production along the French Atlantic coastline since 2001^46^, blue mussel beds are identified as a Habitat of Principle Importance (HPI) under the Natural Environment and Rural Communities (NERC) Act 2006, as a Priority Marine feature (PMF) under the Marine (Scotland) Act 2010, and included on the OSPAR (Annex V) list of threatened and declining species and habitats. This is particularly relevant as summer mortalities of both wild and cultured *M. edulis* have recurrently been reported throughout the world^20,26,47–50^. Noticeably, these aestival mortalities may drastically increase with the expected rise in both mean temperatures and the frequency of extreme high temperature events (IPCC, 2018) warming. In the western Atlantic region, intertidal population of *M. edulis* have experienced catastrophic mortality directly associated with summer high temperatures and, over the last 50 years, a poleward contraction of the species southern range edge has occurred^51^.

**Figure 1:**
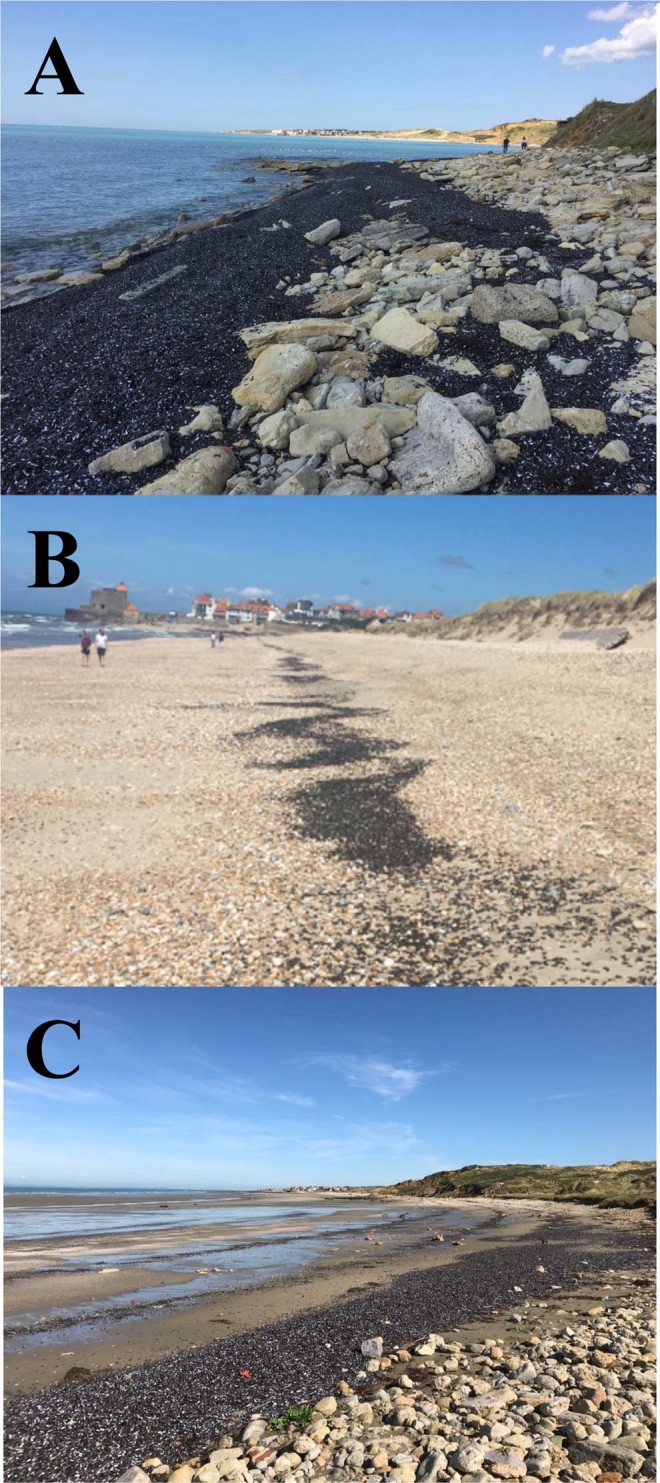
Illustration of the mass mortality of *M. edulis* in the eastern English Channel, where dead empty shells were either found as localised accumulation spots (**A**) on site g shown in Fig. 2, or stretched along kilometres of coastlines as solitary high-tide marks (**B**) or multiple high-tide marks (**C**) respectively found on beaches identified as I and II in Fig. 2.

In this context, we investigate the potential causes and consequences of a mass mortality event of *M. edulis* that occurred along the French coast of the eastern English Channel in early August 2018, when massive quantities of dead empty mussel shells were found washed ashore either accumulated locally (Fig. 1A) or stretched along kilometres of coastlines in one (Fig. 1B) or several high-tide marks (Fig. 1C). Specifically, we used an integrative, climatological and ecophysiological methodology based on three complementary approaches. We first conducted field surveys to assess the size and quantity of dead mussels washed ashore, and to infer the presence of dead mussels on the beds through transects ranging from the upper to the lower limits of *M. edulis* intertidal range. We subsequently inferred the presence of a heat wave and assessed its severity in the study area on the basis of a multidecadal air and sea surface temperature time series. We further examined body temperature temporal patterns in *M. edulis* by continuously recording body temperature *in situ* every 20 minutes using biomimetic mussels. Based on these patterns, we subsequently mimicked the thermal conditions experienced by *M. edulis* during the heat wave in laboratory assays aimed at assessing *M. edulis* lethal temperature. We finally discuss the implications of the heat wave on *M. edulis* in an era of global warming and estimate the financial losses for the local community.

## Methods

### Study area and local climatic conditions

**Figure 2:**
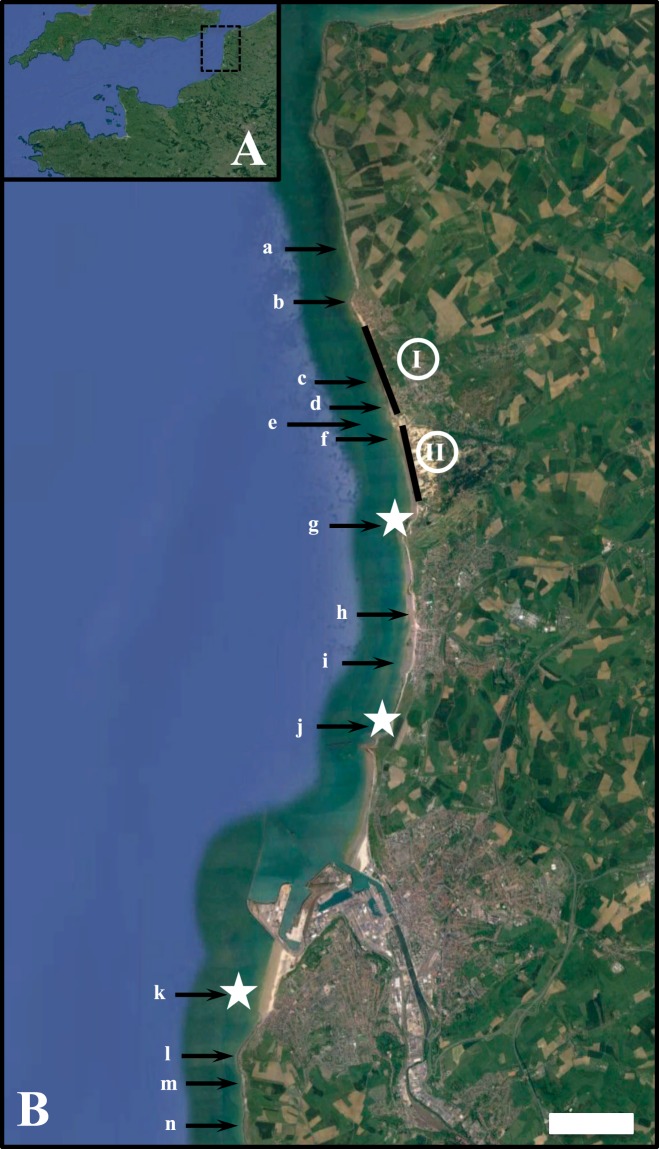
Localisation of the 14 *M. edulis* beds documented along the French side of the English Channel (black arrows), shown together with the accumulation of empty dead shells as localised spots (white stars) and stretched along kilometres of coastlines (black lines). The numbers I and II respectively identify the northernmost and southernmost stretches of beaches where dead *M. edulis* heavily accumulated along the high-tide marks. The scale bar represents 1 km. Map data: Google, SIO, NOAA, U.S. Navy, NGA, GEBCO, Image Landsat/Copernicus (**A**), and Google, SIO, NOAA, U.S. Navy, NGA, GEBCO (**B**).

The present work was conducted along the French coast of the eastern English Channel (Fig. 2). This temperate epicontinental sea is characterised by the amplitude of its semidiurnal tidal range (between 3 and 9 m). The resulting strong (1 to 2 m s^−1^) flood-dominated tidal currents parallel to the coast result in a net flow towards the North sea^52–54^, and generate extremely high levels of mixing, with turbulence intensities ranging from 10^−7^ to 10^−4^ m^2^ s^−3^ ^55^. Sea surface temperature typically ranged between 5 °C in winter and 18 °C in summer, even though surface temperature can be as low as −0.3 °C and as high as 22 °C, and has essentially been dominated by seasonal fluctuations over the last 5 decades^56^. Noticeably, an increase in sea surface temperature has been observed since the mid-1990s^56^ at a rate of ca. 0.4 °C per decade^57^, as well as an increase in the frequency of exceptional events, especially in summer^56^.

This area is also characterised by intertidal rocky reefs and platforms interspersed between stretches of sandy and pebble beaches that are directly exposed to the main SSW swell direction that characterises this area^58^. These intertidal rocky shores host 14 *Mytilus edulis* beds that are all spatially disconnected, essentially by stretches of sandy or pebble beaches with the exception of the three southernmost beds that are separated from the northern ones by two long stretches of sandy beaches and the fishing port of Boulogne-sur-Mer (Fig. 2). These beds range in size from 0.51 to 10.56 hectares, with mussel density ranging from 1,290 to 7,571 mussels per metre square and covering 31.5 to 80.5% of available substrate^59^ (Table 1). Noticeably, the percentage of commercially exploitable mussels is extremely low (i.e. 0.5 to 7%) on all but one of these beds^59^ (Table 1), which jeopardises their sustainability^60^.

**Table 1:** Names and specifics of the 14 *M. edulis* beds known in the eastern English Channel; compiled from Ruellet *et al*. (2016).

Mussel bed	Surface (ha)	Density (ind m^−2^)	>4 cm (%)	Mussel cover (%)
Rupt	0.67	5753	4	0.70
Plats Ridains	6.34	3423	6	0.62
Liettes	4.66	2685	3	0.62
Langues de Chien	4.02	3782	7	0.58
Platier	0.51	2904	3	0.32
Sud de la Slack	2.43	2432	6	0.53
Pointes aux Oies	10.56	1400	6	0.52
Ailettes	3.73	2610	4	0.81
Fort de Croy	2.93	1289	21	0.44
Pointe de la Crèche	9.88	2524	2	0.47
Fort de l'Heurt	5.10	4694	0.6	0.63
Cap d'Alprech	4.59	7571	0.5	0.56
Ningles	2.32	5732	0.8	0.47
Equihen	2.11	5940	0.6	0.44

### Assessing heat wave conditions

The intensity of the thermal forcing observed during the summer 2018 was categorised following Hobday *et al*.^61^, using the climatology of both air and seawater temperature of the study area. Specifically, we categorised the summer 2018 heat wave using multiples of the difference Δ*T* between the climatological mean and the climatological 90^th^ percentile, which is the threshold used to identify marine heat waves (MHW; Hobday *et al*.^62^). The magnitude of this difference was subsequently used as a descriptor of an observed thermal event as moderate (1 to 2 Δ*T*), strong (2 to 3 Δ*T*), severe (3 to 4 Δ*T*) and extreme (>4 Δ*T*) heat waves. The climatology of air and sea surface temperatures were respectively based on hourly air temperature recorded by the Météo France (www.donneespubliques.meteofrance.fr) weather station of Boulogne-sur-Mer (50°43′54N, 1°35′53E) from 1949 to 2017 and biweekly sea surface temperature recorded at the inshore station (50°40′75N, 1°31′17E) of the SOMLIT network (Service d’Observation en Milieu LITtoral; www.somlit.epoc.u-bordeaux1.fr) from 1996 to 2017.

### Field assessment of mussel mortality

On August 17, 18 and 19, we systematically surveyed the 25 kilometres of shoreline between the southernmost and northernmost of the 14 known intertidal mussel beds of the eastern English Channel (Fig. 2, Table 1) to identify where dead mussels were washed ashore as localised accumulation spots or accumulated along high-tide marks (Fig. 1) and subsequently estimate their quantity. Specifically, two observers performed 6 hour surveys each day during daytime low tides. We subsequently identified three localised spots and two stretches of beaches where empty *M. edulis* shells where respectively found forming thick (typically 30 to 60 cm) aggregations at scales ranging from a few metres to tens of metres (Fig. 1A) and in mono- and multi-layered bands accumulated nearly continuously along kilometres of high-tide marks (Fig. 1B,C).

At the three identified localised accumulation sites, we first estimate the surface *S*_*a*_ covered by dead mussels using drone digital photographs (PARROT BEBOP 2) taken from a height of 20 m and previously calibrated using a white disk of PVC (diameter 50 cm). The area covered by mussels was subsequently assessed using the software Image J (https://imagej.nih.gov/ij/). We then estimated the thickness *T*_*i*_ of each accumulation zone from 10 haphazardly chosen points, where 1 litre of dead shells were sampled, their abundance *n*_*i*_ (ind l^−1^) subsequently estimated and each shell length measured with an electronic calliper. The number of dead mussels was estimated as *N*_*a*_ = *S*_*a*_ × *T*_*a*_ × *n*_*a*_, where ${T}_{a}=\frac{1}{10}\mathop{\sum }\limits_{i=1}^{10}{T}_{i}$ is the mean thickness of mussel accumulation and ${n}_{a}=\frac{1}{10}\mathop{\sum }\limits_{i=1}^{10}{n}_{i}$ the mean mussel density per litre.

Where *M. edulis* were found accumulated along high-tide marks, we first estimated the length *L*_*i*_ and width *W*_*i*_ of each high-tide mark *i* from drone digital photographs (see above). We subsequently estimated the density (*d*_*i*_, ind m^−2^) of dead *M. edulis* using *n*_*i*_ 25 × 25 cm quadrats regularly placed on high-tide marks. The number of dead mussels *N*_*i*_ found along each high-tide mark was estimated as *N*_*i*_ = *L*_*i*_ × *W*_*i*_ × *d*_*i*_.

### Assessing *M. edulis* body temperature

To examine body temperature temporal patterns of the blue mussel *Mytilus edulis* at the study sites, we used data from an ongoing survey of the thermal properties of the intertidal ecosystems of the eastern English Channel. Data consisted of *M. edulis* body temperature recorded every 20 minutes from June 6, 2017 using biomimetic mussels (i.e. ‘robomussels’^63^) that were built with empty mussel shells (40–45 mm in length) filled with silicone sealant encasing a temperature logger (Thermochron iButton DS1922L; resolution 0.5 °C). Robomussels were deployed with the anterior-posterior axis perpendicular to their hard rock substratum, in growth position (posterior upward) in intact natural beds using marine grade epoxy resin (Z-spar Splash zone, A-788). Robomussels were deployed within mussel beds, as loggers deployed as solitary individuals tend to yield anomalously high readings^64^. Previous to deployment, robomussel temperature readings (*N* = 20) were tested by placing them in pairs next to live mussels fitted with K-type thermocouple probes (4 Channels Lutron TM-903 Thermometer, resolution 0.1 °C) for aerial temperature ranging from 10 to 40 °C. Readings from robomussels (*T*_*rm*_) and live mussels (*T*_*lm*_) were highly significantly correlated (*r*^2^ = 0.98, *p* < 0.01), and the slope *α* and the elevation *β* of the regression line *T*_*rm*_ = 0.99 *T*_*lm*_ + 0.07 could not statistically be distinguished from theoretical expectations *α* = 1 and *β* = 0, respectively (*p* > 0.05). We also deployed unmodified temperature loggers (DSL1922L iButtons; resolution 0.5 °C) to record rock surface temperature both on bare rocks and under mussel beds. iButtons were wrapped in parafilm, epoxied into shallow depressions chiselled into the rock, and covered by a 1–2 mm layer of epoxy, which was flush with the rock surface.

### Assessing *M. edulis* thermal tolerance

In October 2018, we estimated the thermal limits of *M. edulis* as the temperature lethal to 50% of individuals (LT_50_) following a 6-h aerial exposure. The experiment was designed to mimic the temporal dynamics of *M. edulis* robomussels deployed in the field during the heat wave in terms of (i) rate of temperature increase and decrease, (ii) maximum temperature reached and (iii) duration of exposure. To simulate the two tidal events per day characterising the tidal regime of the eastern English Channel, we used a 18-h recovery period set-up as a 6-h immersion, 6-h aerial exposure and 6-h immersion, all at ambient environmental water and air temperature (i.e. 16 °C). To assess the thermal tolerance limits of the populations, we collected individuals from three beds where local accumulations of dead mussels were found (i.e. Fort de l’Heurt, Pointe de la Crèche and Pointes aux Oies; Fig. 2, Table 1) and assess their thermal tolerance immediately upon return to the laboratory (typically within 30 minutes) to avoid any tolerance changes that may occur during laboratory acclimation^65^.

For each experimental treatment (i.e. 20, 26, 29, 32, 35, 38 and 41 °C), ten mussels were placed in a sealed 1-litre glass jar along with a seawater-saturated paper towel to maintain 100% relative humidity^66^, hence to prevent both any desiccation and evaporative cooling induced by gaping^67^. Replicate jars (*n* = 6, total *N* = 60 mussels for each experimental treatment) were submersed in a water bath for 6 hours, where mussels were first heated at rates representative of the conditions encountered by *M. edulis* in the eastern English Channel (i.e. from 3.5 to 4.5 °C per hour; see below) from ambient seawater temperature (i.e. 16 °C) until one of seven experimental temperatures was reached. This temperature range has been chosen to reflect the temperature patterns experienced by *M. edulis* at our study sites (Seuront, unpublished data), including in July and August (see below), and previously published values of *M. edulis* lethal thermal limits, i.e. typically between 25 to 37 °C^26,68–71^. Experimental temperatures were maintained for 1.5 and 3 hours before being quickly cooled down back to ambient seawater temperature by immerging the jars in running seawater, and thus entering an 18-h recovery period dichotomised as 6-h immersion, 6-h aerial exposure and 6-h immersion at ambient environmental water and air temperature (i.e. 16 °C). The whole procedure was repeated over 5 consecutive days, simulating the daytime thermal aerial exposure event per day.

An additional treatment based on a temperature increase of 1 °C every 5 min followed by a 6-h exposure to each of the six experimental temperatures and a recovery period as described above was also repeated over 5 consecutive days to assess the effect of extremely rapid and severe warming events that have previously been described in temperate intertidal mussel beds^65^ on the thermal tolerance of *M. edulis*.

A robomussel was included within each temperature treatment to monitor the temperature of a real mussel. We used survival in ambient temperature controls where jars were held in a 60-litre aquarium of running natural seawater at 16 °C as our indicator of any handling stress. Survival was assessed after the recovery period via inspection for movement or responsiveness to probing, and we calculated LT_50_ for each site using individual generalised linear models with binomial error distributions, with mussel survival modelled as a function of aerial temperature.

## Results

### Assessing heat wave conditions

Based on a 68-year air temperature climatology and the heat waves (HW) classification^61,62^, four HWs (which lasted between 3 and 5 days) were identified from air temperature in 2018: one in late June (June 28 to 30; 19.2 to 25.7 °C), two in July (July 5 to 7 with 20.3 to 21.9 °C, and July 23 to 25 with 21.9 to 22.8 °C) and one in August (August 1 to 5; 20.4–22.9 °C). In addition, five HWs were identified on June 23 (18.7 °C) and 26 (18.9 °C), July 13 (20.5 °C) and 17 (21.1 °C), and August 10 (20.4 °C). These events were classified as moderate (78.1%) to strong (21.9%) in June, moderate (67.8%), strong (23.1%), severe (7.0%) and extreme (2.0%) in July, and moderate (63.0%), strong (30.9%) and severe (6.2%) in August. In contrast, based on a 21-year sea surface temperature climatology, sea surface temperatures were only marginally considered as moderate heat waves in July and August.

### Assessing mussel mortality

The three local accumulation zones (areas ranging from 45 to 160 m^2^) were covered by layers of dead mussels that were 0.3 to 0.6 m thick. The volume and number of dead mussels was subsequently estimated as ranging from 18 to 96 m^3^ and 3.1 × 10^6^ to 1.4 × 10^7^ mussels, respectively (Table 2). More specifically, based on the percentage of dead mussels of commercial size (i.e. >4 cm in length) found in the accumulation zones (i.e. 21 to 33%), a conservative estimate of 70 fresh commercial mussels per litre, and a volume-to-mass ratio of 1.43 (i.e. one litre of fresh commercial mussel typically weights 0.7 kg), the number and mass of dead mussel of commercial value were respectively estimated as totalling 6.7 × 10^6^ mussels and 67.4 tons (Table 2).

**Table 2:** Surfaces (*S*_*a*_), thickness (*T*_*a*_) and volume (*V*_*a*_) of the three localised accumulation zones of dead empty shells of *Mytilus edulis*, identified with the identification codes (ID) used in Fig. 2, shown together with the related density (*n*_*a*_) and number (*N*_*a*_) of mussels, the percentage of mussels of commercial size (%) and the subsequent estimate of the number (*N*_*c*_) and mass (*M*_*c*_) of dead mussels of commercial size, together with the related financial losses, respectively based on a market value of 4.5€ per kilogram of mussels of commercial size.

	Pointes aux Oies	Pointe de Ia Creche	Fort de I'Heurt	Total
ID	g	j	k	
*S*_*a*_ (m^2^)	160	75	45	300
*T*_*a*_ (m)	0.6	0.3	0.4	—
*V*_*a*_ (m^3^)	96	22.5	18	142.5
*n*_*a*_ (ind l^1^)	143	190	170	—
*N*_*a*_	1.4 10^7^	5.4 10^6^	3.1 10^6^	2.2 10^7^
% (>4 cm)	33	29	21	—
*N*_*c*_ (>4 cm)	4.6 10^6^	1.6 10^6^	6.4 10^5^	6.7 10^6^
*M*_*c*_ (>4 cm) (ton)	45.3	15.7	6.4	64.7
Losses (€)	203,860	70,666	28,917	303,444

Along the northernmost and southernmost stretches of beaches where *M. edulis* accumulated along high-tide marks (Fig. 2B,C), we respectively identified 2 and 4 high-tide mark accumulation stretching along ca. 3 km of shoreline. These accumulations ranged from 70 to 975 m in length, 0.5 to 6.5 metres in width, and contained from 432 to 6,400 individuals per square metre, representing a total of 4.3 × 10^6^ to 9.2 × 10^6^ dead mussels (Table 3). Given the observed percentage of mussels of commercial size in these high-tide mark accumulations (28 to 34%), the quantity of dead mussels of commercial value ranged between 1.3 × 10^6^ and 2.8 × 10^6^ mussels. Finally, using an estimate of 70 fresh mussels per litre and a volume-to-mass ratio of 1.43, the mass of dead mussels of commercial value was estimated as ranging between 12.8 and 28.5 tons (Table 3).

**Table 3:** Length (*L*_*i*_), width (*W*_*i*_), mussel densities (*d*_*i*_) and abundance (*N*_*i*_) of dead empty shells of *Mytilus edulis* found as mono- and multi-layered bands accumulated nearly continuously along high-tide marks *I* found along the two stretches of beaches identified as I and II in Fig. 2, and estimated from *n*_*i*_ quadrats of 25 cm × 25 cm, shown together with the percentage (%), number (*N*_*c*_) and mass (*M*_*c*_) of mussels of commercial size. The related minimum and maximum financial losses, respectively based on a market value of 4.5€ per kilogram of mussels of commercial size are also provided.

*i*	Beach	*L*_*i*_	*W*_*i*_	*n*_*i*_	*d*_*i*_(ind m^−2^)		*N*_*i*_		%(>4 cm)	*N*_*c*_(> 4 cm)		*M*_*c*_(ton) (>4 cm)		Loss (€)	
					Min	Max	Min	Max		Min	Max	Min	Max	Min	Max
1	II	70	4	8	5600	6400	1.6 10^6^	1.8 10^6^	29	4.5 10^5^	5.2 10^5^	4.5	5.2	20,462	23,386
2	II	250	1	20	992	3200	2.5 10^5^	8.0 10^5^	28	6.9 10^4^	2.2 10^5^	0.7	2.2	3,125	10,080
3	II	250	2	18	544	3056	2.7 10^5^	1.5 10^6^	32	8.7 10^4^	4.9 10^5^	0.9	4.9	3,917	22,003
4	II	150	6.5	12	432	2400	4.2 10^5^	2.3 10^6^	34	1.4 10^5^	8.0 10^5^	1.4	8.0	6,444	35,802
5	I	800	0.5	30	1125	1749	4.5 10^5^	7.0 10^5^	28	1.3 10^5^	2.0 10^5^	1.3	2.0	5,672	8,817
6	I	975	1	30	1360	2133	1.3 10^6^	2.1 10^6^	30	4.0 10^5^	6.2 10^5^	4.0	6.2	17,901	28,080
							**4.3 10**^**6**^	**9.2 10**^**6**^		**1.3 10**^**6**^	**2.8 10**^**6**^	**12.8**	**28.5**	**57,521**	**128,167**

### Assessing *M. edulis* body temperature using biomimetic loggers

**Figure 3:**
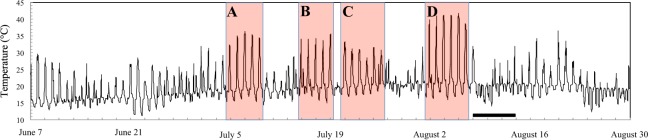
Time series of robomussel temperature from June 7 to August 30, 2018 taken at a 20-min temporal resolution. The colored areas identify the four thermal events identified as periods of 5 to 6 consecutive days with temperatures reaching more than 30 °C (and occasionally 35 °C; (**A–C**) and more than 40 °C (**D**). The black bar identifies the periods when dead empty mussel shells were washed ashore.

Over the 2 months that preceded the observed mass mortality along the shores of the eastern English Channel, four main thermal events were identified from robomussel temperatures as periods of 5 to 6 consecutive days with body temperatures reaching more than 30 °C and occasionally more than 35 °C from July 5 to 9 (32 to 35.9 °C), July 15 to 19 (33.5 to 35.3 °C), July 21 to 26, (30.1 to 33.2 °C), and more than 40 °C (38.2 to 41.7 °C) from August 2 to 6 (Fig. 3). These events were consistently characterised by a 3.5 to 4-h increase in temperature at a 4.2 to 4.3 °C per hour in July, and a 4 to 4.5-h increase in temperature at a rate of 4.4 to 4.6 °C per hour in August. Mussel body temperature reached maximum values ranging between 30.1 and 35.3 °C for 1.3 to 1.5 hours in July and between 38.2 and 41.7 °C for 3 hours in August. The incoming tides subsequently led to a sharp decrease in *M. edulis* body temperature down to seawater temperature at a rate of 12.6 °C per hour in July and 17.5 °C per hour in August.

No significant correlations were found between robomussel temperature and air temperature (Spearman’s *ρ*, *p* > 0.05). In turn, robomussel temperature was significantly positively correlated with the surface temperature of bare rock and the rocks below the mussel beds (Spearman’s *ρ*, *p* < 0.01). Specifically, during the four thermal events described above, robomussel temperatures were consistently highly significantly warmer (on average 9.8 to 17.1 °C warmer; Wilcoxon-Mann-Whitney *U*-test, *p* < 0.01) than air temperature. Robomussel temperatures were also consistently highly significantly warmer (Wilcoxon-Mann-Whitney *U*-test, *p* < 0.01) than bare rocks and the rock under mussel beds, i.e. robomussels were on average 4.5 to 6.2 °C and 11.5 to 13.7 °C warmer than bare rock and the rocks below the mussel beds, respectively.

### Assessing *M. edulis* thermal tolerance

**Figure 4:**
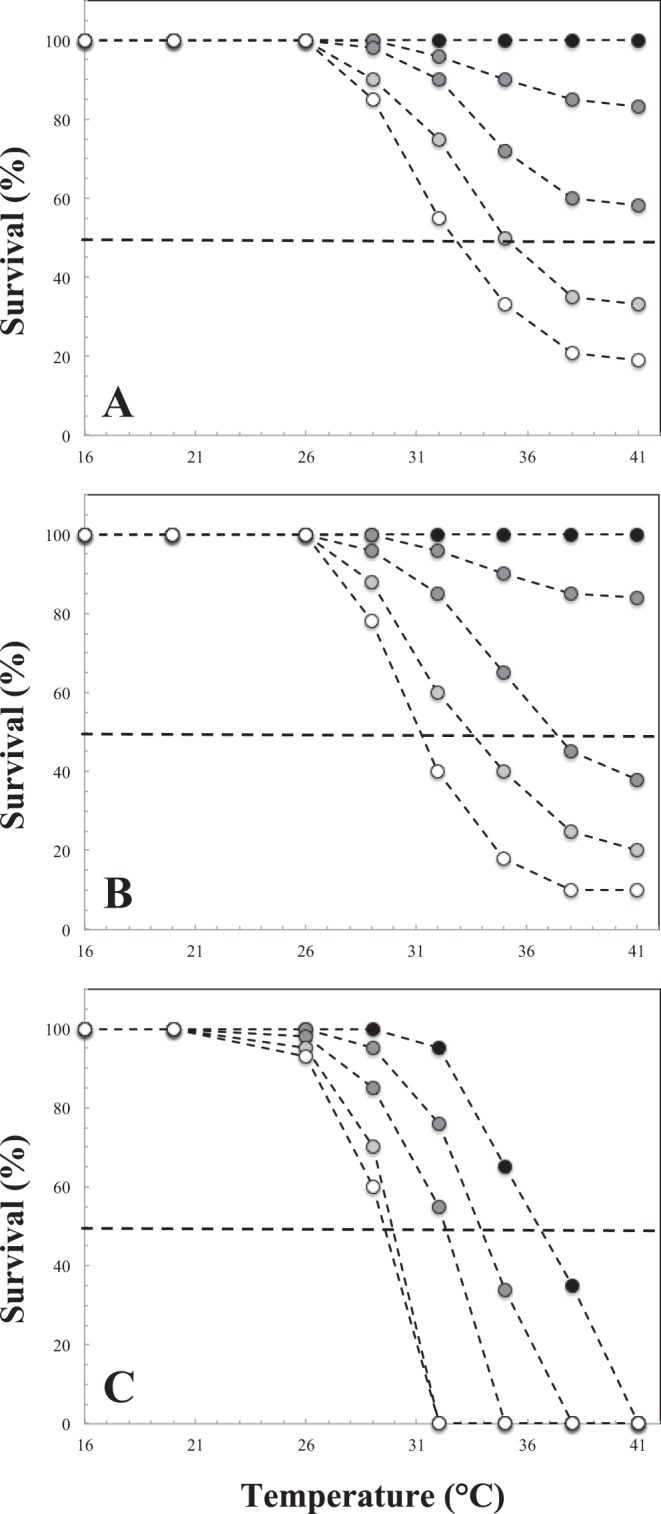
Survival rates (%) of *M. edulis* as a function of experimental temperature for thermal exposure of 1.5 hour (**A**), 3 hours (**B**) and 6 hours (**C**), where each separate curve is a separate exposure event. Black dots: first exposure, dark grey dots: second exposure; intermediate grey dots: third exposure; light grey dots: fourth exposure; white dots: fifth exposure. The dashed horizontal line represents a 50% survival rate.

We examined the effect of temperature, duration of exposure and number of repeated exposures on *M. edulis* mortality through the relationship between the percentage of survival and experimental temperature for each exposure event and duration (Fig. 4). Mussels exposed to control conditions at 16 °C for 1.5, 3 and 6 hours consistently experienced 100% survival, irrespective of the number of repeated exposures. This observation indicates the absence of any physiological impairment related to handling stress.

No mortality was ever observed in mussels exposed only once to temperatures ranging from 20 to 41 °C during 1.5 and 3-h exposure (Fig. 4A,B). In turn, survival consistently decreased at all temperatures above 29 °C, at rates increasing with the number of exposures. Survival rates never reached 0%, though they were consistently higher after a 1.5-h than a 3-h exposure, with minima of respectively 19% and 10% observed after 5 consecutive daily exposure events at 41 °C (Fig. 4A,B). In contrast, a dramatic decline in survival rates was observed after a single 6-h exposure for temperature greater than 29 °C, and mussels exposed only once to 41 °C suffered a 100% mortality (Fig. 4C). With increases in the number of consecutive daily exposure events, 100% mortality occurred at lower temperatures.

**Figure 5:**
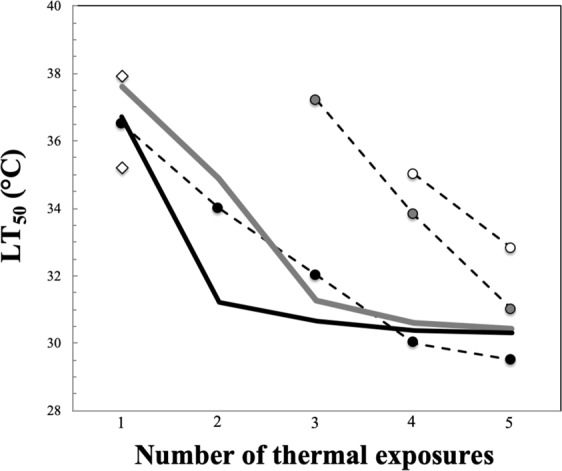
Thermal limits of *M. edulis*, estimated as the temperature lethal to 50% of individuals (LT_50_), shown as a function of the number of successive thermal exposures for thermal exposures of 1.5 hours (open dots), 3 hours (grey dots) and 6 hours (black dots). The two open rhombs are the upper LT_50_ values reported in Sorte *et al*. (2019) and the black and grey lines the LT_50_ reported by Jones *et al*. (2009) respectively in June and November.

The temperature lethal to 50% of individuals (LT_50_) could only be estimated for the fourth and fifth successive 1.5-h thermal exposure, and third, fourth and fifth successive 3-h thermal exposure as the related survival rates did not decrease below 50% for the first 3 and 2 consecutive exposures, respectively (Fig. 4A,B). LT_50_, however, consistently declined with the number of successive daily exposures (Fig. 5). Specifically, the LT_50_ decreased from 36.5 °C with one exposure to an asymptote of ca. 30 °C after 3 to 4 consecutive daily exposures (Fig. 4). No significant differences in survival rates calculated as a function of temperature for any of the thermal exposure considered were observed between sites (Kruskal Wallis *H* test, *p* > 0.05).

## Discussion

### *M. edulis* body temperature and local climatology

*M. edulis* body temperatures were consistently significantly higher than air temperature (*p* < 0.01), and no significant correlation was found between *M. edulis* body temperature and air temperature (*p* > 0.05). These observations are consistent with previous studies highlighting the fact that the body temperature of ectothermic poikilotherms, either intertidal or terrestrial, are heavily constrained by the their exposure to direct solar radiation and the structure of their microhabitat, such that their temperatures are only close to air temperature in fully shaded microhabitats^72,73^. More specifically, despite the moderate nature of both the atmospheric and marine heat waves observed before the mass mortality took place, the body temperatures observed in the present work (i.e. 30.1 to 41.7 °C) are in the high range of mussel body temperature reported in the literature^74–76^. These results are consistent with previous observations conducted on tropical and temperate intertidal gastropods^77–82^ and confirm that environmental and individual body temperatures are decoupled. They also generalize to previous work on *M. edulis* that showed that body temperature of intertidal gastropods is primarily controlled by non-climatic heat sources, i.e. solar irradiance^77,83^ instead of climatic heat sources, i.e. air and water temperatures^77,84,85^. In addition, biotic factors such as shell morphology, surface colour and patterning and thermal properties (i.e. heat transmission, absorption, reflection and conduction) are also very likely to significantly affect body temperature^31,76,86,87^.

In a more general context, these results suggest that climate change models based on air temperature as a proxy for body temperature are likely to underestimate the effect of global warming on the body temperature of intertidal species and subsequently overestimate their physiological tolerance, and with consequences on scenarios of future species distribution range^72^. The mechanistic links between the body temperatures of ectotherms, which control local^88^ and global distribution patterns^89^, and environmental variables are not as simple as previously anticipated^90,91^. This issue is especially critical for intertidal ectotherms, especially for sessile species such as mussels, which have a limited ability to buffer the effect of thermal stress through behavioural adaptation and active selection of thermally benign habitats^82,92^, as they often live close to the upper edge of their thermal window^93^. A thorough assessment of their thermal tolerance is hence needed to anticipate population level effects of heat stress and more generally change in distribution patterns, local zonation and biogeography^21,94,95^.

### *M. edulis* body temperature and substrate temperature

*M. edulis* body temperatures (i.e. 30.1 to 41.7 °C) were on average 4.5 to 6.2 °C warmer than the surrounding back rocks, and 11.5 to 13.7 °C warmer than the rocks below the mussel beds. These results are consistent with measurements conducted across *M. californianus* beds^96^. This directional shift in temperature modification has been shown to influence interactions with juvenile mussels, such that thermal stresses and associated mortality risk are higher at the bed surface, but substantially reduced deeper within the adult matrix^96^. Mussel beds are key ecosystem engineers that increase habitat complexity. They consequently provide substrate for colonization, trap sediment and organic particles that serve as food for small invertebrates and shelter from predation^33–35^. They also facilitate the establishment and persistence of a range of small invertebrates, hence enhance local biodiversity^38,39^ and provide protection against wave-action^32^. Despite increasing evidence that mussel beds create microhabitats that remain moist and thermally benign during low tides^30,31,96^, their role in dampening thermal stress and eventually maintaining local biodiversity during heat waves under a global change scenario is still a relatively untapped area of research. This issue is of paramount importance as mussel beds are globally acknowledged as biodiversity hotspots^97–99^, including in the eastern English Channel, where intertidal mussel beds host up to 62 taxa of epifauna^60,100^. Note, however, that evidence exists on the decline of mussel bed community diversity as a response to decadal climate change^101^. The increase in local thermal heterogeneity related to mussel beds is consistent with numerous studies conducted on the thermal properties of intertidal rocky substrates providing evidence that variation over very small scales can be equivalent or even exceed mean differences observed over much larger scales^102–104^. For example, the difference in body temperatures between the warmest and coolest mussels over an area of a few square meters (up to 15 °C on any given day) rivalled and sometimes greatly exceeded the expected difference in body temperatures along ca.1600 km of rocky intertidal zone of the western coastline of North America^102^. Further work is needed, however, to quantify how small-scale thermal heterogeneity, including the thermal heterogeneity observed across and within mussel beds, may dampen thermal stress and affect the resilience of local survival and biodiversity in an era of global warming where both the intensity and severity of heat waves are expected to increase^4–6^.

### *M. edulis* thermal tolerance: the role of consecutive exposures

Much attention has been given to the thermal tolerance of mussels during immersion as a geographic range limiter^57,68,105,106^, mainly because there is no escaping from high water temperatures. For instance, Wells & Gray^106^ suggested that mean summer water temperatures of 26.7 °C set the southern range limit, whereas Hutchins^105^ believed the southern limit to be set by winter isotherms of 8 °C. Recent work showed that the hybrid zone between warm- and cold-adapted species (*M. galloprovincialis* and *M. edulis*, respectively) will move eastward into the English Channel towards the territory formerly occupied by the cold-adapted species in response to a warming climate^57^. In contrast, the detrimental effects of high temperatures during emersion may be alleviated through local changes in distribution patterns such as local zonation^21,94,95^ and aggregation patterns^74^. High temperatures experienced during emersion at low tide have nevertheless the potential to cause high rates of mortality^20,25–27^, which triggered studies devoted to elucidate the impact of aerial exposure on survival^20,21,65,85,95,107^.

Though the thermal limits of mussels in water are the ones demonstrating plasticity and local selection^70,71,108,109^, we specifically focused on *M. edulis* thermal limits during emersion as the seawater temperatures observed in the eastern English Channel (typically bounded between 16 °C and 20 °C in summer^56^) are consistently well below the temperature causing death in this species, which ranged between 25 °C and 41 °C^68,70,71,108,110,111^. The population of *M. edulis* of the eastern English Channel is very resistant to a single 1.5-h and 3-h exposure to temperature ranging from 20 to 41 °C, with no recorded mortality (Fig. 4A,B). With the exception of repeated aerial exposures to 16 °C (control), 20 °C and 26 °C treatments, repeated exposures to temperatures of 29 to 41 °C decreased survival rates (Fig. 4A,B). In contrast, a single 6-h exposure to 41 °C led to 100% mortality, and repeated exposure led to 100% mortality for temperatures ranging from 32 to 41 °C (Fig. 4C). Taken together, and irrespective of the exposure duration, the effects on population mortality of repeated exposures to less severe temperatures over a 3 to 5-day period are as severe as a single exposure to very high temperatures, indicating that multiple exposures decrease thermal tolerance.

The thermal limits of *M. edulis*, estimated as the temperature lethal to 50% of individuals (LT_50_) following an aerial exposure of 1.5, 3 and 6 hours to temperatures ranging from 20 to 41 °C are in the range of values reported for *M. edulis* (Fig. 5) following a single 6-h exposure to aerial thermal stress ranging from 23 to 45 °C^71^ and from 30 to 37 °C^65^. The increase in LT_50_ observed for a given number of repeated daily exposures with decreasing exposure duration (Fig. 5) finally suggests that the thermal tolerance of *M. edulis* to repeated heat stress events is likely to be higher towards the lower limits of their tidal range, where they spend less time out of the water. This result is particularly important for the sustainability of the species in this area, especially in an era of global warming characterised by increasing intensity and severity of heat waves. It also has potential critical implications for both the current attempts to expand mytiliculture in the eastern English Channel and the development and implementation of management and conservation strategies of the local natural mussel beds, which are of patrimonial and commercial relevance.

Our results are also consistent with observations resulting from successive daily exposure of *M. edulis* to 6-h aerial thermal stress ranging from 23 to 45 °C^71^, and a study on *Littorina littorea* heat coma showing that the temperature at which heat coma occurred declined significantly with repeated daily exposures^112^. They contrast, however, with previous work on the thermal tolerance of mussels^113^ and corals^114^, which suggests that thermotolerance conferred upon exposure to deleterious temperatures allows organisms to tolerate or even acclimate to continued exposures, with a subsequent reduction in mortality rate compared to the initial event. While the resolution of this discrepancy is beyond the scope of the present study, and may be related to differences in the frequency of the thermal exposures, thermal history and/or species-specific induction of heat shock proteins, the fact that multiple exposures decrease the thermal tolerance in *M. edulis* suggests that in a context where the intensity of heat events are expected to increase in intensity and frequency^4–6^, this population may be increasingly at risk. This is particularly critical in the eastern English Channel, where the documented low (i.e. 0.5 to 7%) frequency of large mussels (i.e. >4 cm) on most mussel beds^59^ (Table 1), already jeopardises their sustainability^60^.

### Implications for the local economy

Based on the conservative estimates of the quantity of dead mussels of commercial size (i.e. >4 cm) found in both the local accumulation zones and along high-tide marks, using an average retail price of 4.5€ per kilogram of fresh mussel^45^, the direct economic losses associated to the dead mussels of commercial size found either in localised accumulations or stretched along high-tide marks respectively represent ca. 300,000€ (Table 2) and between ca. 57,000 and 128,000€ (Table 3), totalling between 357,00 and 428,000€. Because mussel of commercial size represented only ca. 30% of the dead mussels found in both local aggregations (21–33%) and along high-tide marks (28–34%), the indirect economic losses related to the death of mussels that would not reach the commercial size ranged between 1,120,000 and 1,430,000€. Overall, the combination of direct and indirect losses are in the range 1,550,000 and 1,860,000€.

Finally, to put these figures in perspective in a local economical context, the amount of mussels taken annually from the natural mussel beds of the eastern English Channel by recreational and professional fisheries are respectively 250 and 450 tons^44,45^, representing a net worth of 1,200,000 and 2,000,000€. The mass mortality reported in the present study then represents a net loss ranging between 138 and 165% and 77 and 92% of the annual commercial value of recreational and professional fisheries, respectively. Overall, the net loss related to this unique mussel mortality event ranges between 49 to 59% of the annual commercial value of recreational and professional fisheries combined.

## Conclusion

Our results indicate that the mass mortality of the blue mussel *Mytilus edulis* reported in summer 2018 in the eastern English Channel occurred under relatively moderate heat wave conditions. This observation indicates that *M. edulis* body temperature is controlled by non-climatic heat sources instead of climatic heat sources, as previously reported for intertidal gastropods. More fundamentally, our results indicate that multiple exposures drastically decreased thermal tolerance, hence increased population mortality. This is particularly critical in an era of global change where heat events are expected to increase in intensity and frequency, especially in the eastern English Channel where the low frequency (0.5 to 7%) of commercially exploitable mussels (i.e. >4 cm) questions both their ecological and commercial sustainability^60^. The present work then raised the question of the future sustainability of local mussel beds, and stress the need for the development and implementation of management and conservation strategies. This is especially critical as beyond their role in maintaining ecosystem structure and function and their economic value, mussel beds are increasingly recognised for their social and patrimonial value. More generally, our results stress the risk of losses in ecosystem integrity and services of intertidal communities under the influence of climate change^115^.

## Data Availability

The datasets generated during and/or analysed during this study can be obtained from the corresponding author by reasonable request.
